# Interleukin-12 family cytokines and sarcoidosis

**DOI:** 10.3389/fphar.2014.00233

**Published:** 2014-10-27

**Authors:** Sabine Ringkowski, Paul S. Thomas, Cristan Herbert

**Affiliations:** ^1^Inflammation and Infection Research Centre, Faculty of Medicine, University of New South WalesSydney, NSW, Australia; ^2^Respiratory Medicine Department, Prince of Wales HospitalSydney, NSW, Australia; ^3^Faculty of Medicine, University of HeidelbergHeidelberg, Germany

**Keywords:** sarcoidosis, IL-12, IL-23, IL-27, IL-35, pathogenesis, granuloma, anergy

## Abstract

Sarcoidosis is a systemic granulomatous disease predominantly affecting the lungs. It is believed to be caused by exposure to pathogenic antigens in genetically susceptible individuals but the causative antigen has not been identified. The formation of non-caseating granulomas at sites of ongoing inflammation is the key feature of the disease. Other aspects of the pathogenesis are peripheral T-cell anergy and disease progression to fibrosis. Many T-cell-associated cytokines have been implicated in the immunopathogenesis of sarcoidosis, but it is becoming apparent that IL-12 cytokine family members including IL-12, IL-23, IL-27, and IL-35 are also involved. Although the members of this unique cytokine family are heterodimers of similar subunits, their biological functions are very diverse. Whilst IL-23 and IL-12 are pro-inflammatory regulators of Th1 and Th17 responses, IL-27 is bidirectional for inflammation and the most recent family member IL-35 is inhibitory. This review will discuss the current understanding of etiology and immunopathogenesis of sarcoidosis with a specific focus on the bidirectional impact of IL-12 family cytokines on the pathogenesis of sarcoidosis.

## INTRODUCTION

The term sarcoidosis was first used in 1899 to describe pathological features of skin lesions, but is currently used to describe a systemic granulomatous inflammatory disease predominantly affecting the lungs (Boeck, 1899; Müller-Quernheim et al., 2012). The non-caseating granulomas observed in affected tissues remain its key pathological feature (Heinle and Chang, 2014). More than 100 years of research have been unable to reveal the etiology and pathogenesis of the disease.

Current investigations into the pathogenesis include studies of gene polymorphisms and the role of possible infective and non-infective antigens (Adrianto et al., 2012; Negi et al., 2012; Dubaniewicz et al., 2013). Important questions that remain unexplained are how and why granulomas are formed and why approximately 20% of all patients develop pulmonary fibrosis whereas the majority experience remission (Iannuzzi and Fontana, 2011; Müller-Quernheim et al., 2012; Broos et al., 2013; Loke et al., 2013). Also yet to be explained is the observed anergy of peripheral T-cells in affected patients (Lee et al., 2011).

The IL-12 family of cytokines, (IL-12, IL-23, IL-27, and IL-35), have been implicated in other granulomatous inflammatory diseases such as tuberculosis and Crohn’s disease, and a role for some of these cytokines has also been proposed in sarcoidosis (Larousserie et al., 2004; Mroz et al., 2008; Judson et al., 2012). Thus the purpose of this review is to discuss the current understanding of the etiology and pathogenesis of sarcoidosis with a focus on a possible role for the IL-12 family cytokines.

## EPIDEMIOLOGY

The incidence and prevalence of sarcoidosis varies between different ethnic groups. Sarcoidosis is more common in females and peak incidence is between 30–50 years (Rybicki and Iannuzzi, 2007). African Americans and Northern Europeans have the highest incidence rate ranging between 15–80/100.000 (Rybicki and Iannuzzi, 2007). Interestingly more recent studies in different countries all reveal an increased prevalence and incidence compared to former reports, indicating that sarcoidosis might be more common than previously thought (Deubelbeiss et al., 2010; Nicholson et al., 2010; Erdal et al., 2012).

## ETIOLOGY

Sarcoidosis is believed to be caused by exposure to antigens and environmental agents in genetically susceptible individuals (Eishi, 2013).

The ACCESS study *(A case control etiologic study of sarcoidosis)* identified exposure to insecticides as well as mold, mildew, and musty odors as risk factors pointing towards a role of microbial bioaerosols in the pathogenesis of sarcoidosis (Newman et al., 2004). The same study confirmed a significantly higher risk for first and second degree relatives of affected patients to be diagnosed with sarcoidosis suggesting an involvement of genetic factors (Rybicki et al., 2001).

### POTENTIAL ANTIGENS

Several observations support the idea of microbial antigens playing a role in the pathogenesis of sarcoidosis. Early studies found that tissue samples from sarcoid patients injected into animals caused granuloma formation (Iwai and Takahashi, 1976; Mitchell et al., 1976). However when similar samples are disinfected they do not cause granuloma formation, suggesting a cell-mediated or microbial origin (Ikonomopoulos et al., 2000, 2006). Similarly several case reports indicate that sarcoidosis might be transmittable via organ transplantations (Burke et al., 1990; Heyll et al., 1994; Padilla et al., 2002; Pukiat et al., 2011; Das et al., 2012). Likewise, there are reports of both successful antibiotic and antifungal treatment of sarcoidosis perhaps related to the potential sarcoid antigens, *Mycobacterium tuberculosis, Propionibacterium acnes,* and more recently, fungi (Terčelj et al., 2007, 2011a; Drake et al., 2013; Takemori et al., 2014).

As sarcoidosis is a granulomatous disease, *M. tuberculosis* has long been suspected to be involved, yet the bacterium has never been isolated from sarcoid tissue (Milman et al., 2004). Nonetheless newer methods of detection have revealed that *M. tuberculosis* antigens are present in sarcoid lesions (Gupta et al., 2007; Oswald-Richter et al., 2012). Peripheral blood mononuclear cells (PBMCs) as well as bronchoalveolar lavage fluid (BAL) cells show hypersensitivity when stimulated with those antigens, producing higher amounts of interferon gamma (IFNy) than healthy controls without Bacillus Calmette-Guerin vaccination (Oswald-Richter et al., 2009, 2012; Ahmadzai et al., 2012).

Similarly, *P. acnes* antigens cause hypersensitivity in only a subgroup of patients but the bacterium is detected in and isolated from sarcoid lymph nodes and tissue more frequently than in healthy controls (Furusawa et al., 2012; Negi et al., 2012).

Fungal exposure is another risk factor for sarcoidosis and higher levels of beta-glucan (a fungal cell wall component) have been found in BAL fluid of patients compared to controls suggesting a possible role for fungal antigens in sarcoidosis (Newman et al., 2004; Terčelj et al., 2011b, 2013).

Despite the evidence for involvement of both *M. tuberculosis* antigens and *P. acnes* as well as fungal exposure in the pathogenesis of sarcoidosis, these organisms only amount for a subgroup of patients leaving room for other theories that suggest a role for non-microbial antigen such as autoantigens, serum amyloid A, and human heat shock proteins (Salazar et al., 2000; Wahlstrom et al., 2007, 2009; Chen et al., 2010; Bargagli et al., 2011; Dubaniewicz, 2013; Zhang et al., 2013).

### GENE POLYMORPHISMS

Just as there is probably no single “sarcoid antigen,” there is no single “sarcoidosis gene.” Multiple gene polymorphisms associated with sarcoidosis have been identified in different regional subgroups but many of the results can not be reproduced in different cohorts (Spagnolo and Grunewald, 2013). Human leucocyte antigen (HLA) polymorphisms have received the most attention with the hypothesis that sarcoidosis might be an antigen driven disease. Whilst HLA-DRB1^∗^03 is associated with an increased risk for Löfgren’s syndrome, HLA-DRB1^∗^07, ^∗^11, ^∗^14, and ^∗^15 are related to chronic disease while HLA^∗^DRB^∗^01 and ^∗^13 seem to be protective (Grunewald et al., 2010; Sato et al., 2010; Wijnen et al., 2010; Grubic et al., 2011; Zhou et al., 2011; Wennerstrom et al., 2012). Only two non-HLA polymorphisms have been confirmed: annexin A11 being protective whereas a butyrophilin-like 2 polymorphism is associated with chronic disease (Spagnolo et al., 2007; Milman et al., 2011; Wijnen et al., 2011; Adrianto et al., 2012; Morais et al., 2012; Suzuki et al., 2012). More recently, genome-wide association studies and single polymorphism analyses have also suggested a role for toll-like receptors, the myeloid differentiation primary response gene (Judson et al., 2012), IL-23 receptor (IL-23R), TNF-α, IL-10, *NOTCH4*, and *OS9* polymorphisms (Veltkamp et al., 2007; Vasakova et al., 2010; Kim et al., 2011; Adrianto et al., 2012; Daniil et al., 2013; Hofmann et al., 2013; Song et al., 2014; Wijnen et al., 2014). As yet, none of these findings have been shown to have external validity and their potential clinical significance and contribution to the pathogenesis remain to be determined. Thus the literature shows the concept of sarcoidosis being an antigen-driven immunoreaction in genetically susceptible individuals to be accurate in principle but lacking in specificity in all patient groups.

## PATHOGENESIS

### INITIATION, ACCUMULATION, AND EFFECTOR PHASE

**FIGURE 1:**
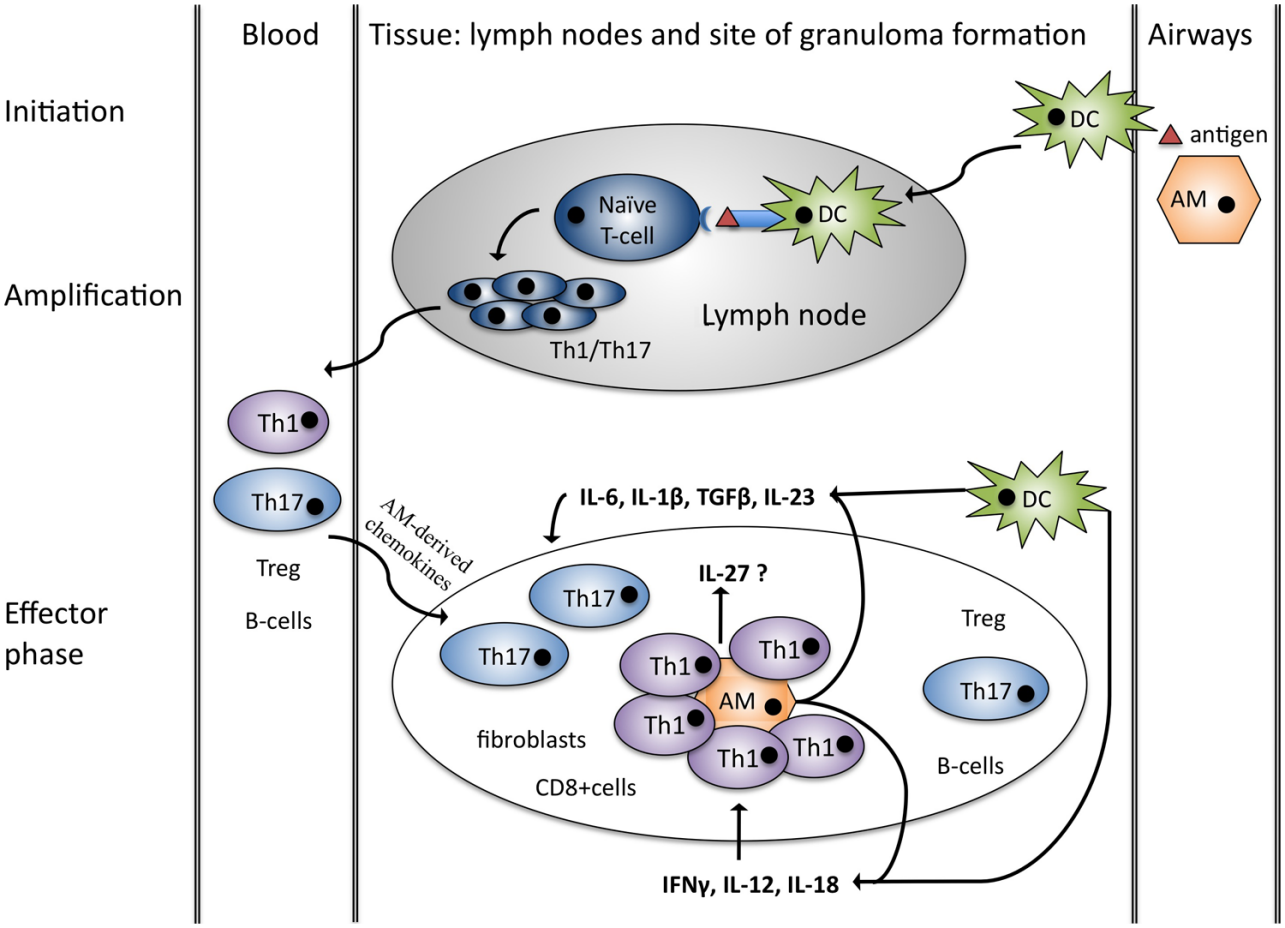
**Granuloma formation – Th1/Th17 hypothesis.** Initiation: alveolar macrophages (AM) and dendritic cells (DC) are activated by a putative antigen. DC migrates to lymph nodes and initiates Th1/Th17 cell amplification. Chemokines produced by alveolar macrophages attract Th1/17, Treg, B-cells as well as CD8+ cells and fibroblast and initiate granuloma formation (effector phase). Both DC and macrophages produce cytokines favoring Th1 and Th17 cells in sarcoidosis. Figure adopted from Broos et al. (2013).

Sarcoidosis is characterized by non-caseating granulomas that typically consist of a core of Th1 cells and activated macrophages surrounded by B-cells, fibroblasts and CD8 lymphocytes as well as Th17 cells, and Treg cells (Miyara et al., 2006; Iannuzzi and Fontana, 2011; Ten Berge et al., 2012). Granuloma formation can be explained in four stages: initiation, accumulation, effector phase, resolution/development of fibrosis. It has traditionally been explained by antigen-driven Th1 responses and interactions between antigen presenting cells (APC) and Th1 cells but recent advances have implicated Th17 in the process of granuloma formation as well (**Figure 1**; Co et al., 2004; Chen and Moller, 2011; Iannuzzi and Fontana, 2011; Müller-Quernheim et al., 2012; Broos et al., 2013).

Th1 cell accumulation in the lungs is characteristic of sarcoidosis and these cells spontaneously produce increased amounts of IL-2 and IFNγ in the BAL fluid (Robinson et al., 1985; Moseley et al., 1986; Prasse et al., 2000). The development of these Th1 cells depends on IL-12 and IL-18, both of which are elevated in the BAL fluid of sarcoidosis patients supporting a Th1-based hypothesis of granuloma formation (Stoll et al., 1998; Yoshimoto et al., 1998; Shigehara et al., 2001; Mroz et al., 2008).

Increased numbers of Th17 cells in BAL, blood and granulomatous tissue of sarcoidosis patients suggest a contribution of these cells in the pathogenesis of sarcoidosis (Facco et al., 2011; Ten Berge et al., 2012; Richmond et al., 2013). Th17 cells are a subset of pro-inflammatory CD4+ cells that are associated with autoimmunity and antimicrobial defense (Bedoya et al., 2013). They develop from naïve CD4+ T-cells in the presence of TGFβ, IL-1β, IL-6, and IL-23 (Acosta-Rodriguez et al., 2007; Wilson et al., 2007; Manel et al., 2008; Volpe et al., 2008). In patients with sarcoidosis IL-1β, IL-6, and TGFβ are elevated in the BAL fluid compared to healthy controls and IL-23 has been detected in granulomas creating a Th17 supportive microenvironment (Zissel et al., 1996; Idali et al., 2006; Judson et al., 2012; Urbankowski et al., 2012). A few studies have analyzed the role of the Th17 effector cytokines IL-17A/F, IL-21, and IL-22 in sarcoidosis, but the results are contradictory. One group demonstrated that sarcoid Th17 cells produce more IL-17A compared to healthy controls, whereas others demonstrated that IL-17 was down-regulated after stimulation with *P. acnes* (Furusawa et al., 2012; Richmond et al., 2013). In skin lesions of sarcoidosis patients, IL-21 has been found to be elevated, whereas IL-17 and IL-22 were not dysregulated (Judson et al., 2012). Thus there is some evidence for a role of Th17 cells in granuloma formation but more studies are needed to define the role of Th17 effector cytokines.

### RESOLUTION VERSUS FIBROSIS

The factors determining the clinical course of sarcoidosis, either granuloma resolution or progression to fibrosis, remain unclear (Patterson et al., 2012). On a cellular level remission is believed to occur through antigen clearance through a strong Th1 response and recovery of regulatory T-cells (Treg; Müller-Quernheim et al., 2012; Oswald-Richter et al., 2013). There is evidence supporting the theory that fibrosis is caused by a switch from Th1 responses towards Th2 (Iannuzzi et al., 2007; Müller-Quernheim et al., 2012). In line with this hypothesis, the concentration of the Th2 cytokine IL-5 is lower in those without fibrosis compared to those with fibrotic disease (Patterson et al., 2013). Further support for a switch to Th2 responses is provided by several studies that identified a Th2 driven immunosuppressive polarization of alveolar macrophages (AM) in fibrotic patients although these findings remain controversial (Prasse et al., 2006; Wikén et al., 2010; Prokop et al., 2011). Thus there is some evidence suggesting a contribution of Th2 responses to the development of fibrosis but further studies are required to confirm this theory. It may also be worth exploring a potential role of other immunosuppressive cytokines such as IL-27 and IL-35.

### IMMUNE PARADOX AND PERIPHERAL ANERGY

T-cell anergy is a term to describe hypo-responsiveness or incomplete activation of T-cells after exposure to common recall antigens, thought to be associated with a lack of adequate co-stimulation or overexposure to co-inhibitory signals (Crespo et al., 2013). In sarcoidosis, anergy is used to describe a lack of reaction to skin antigen tests (delayed type hypersensitivity) and *ex vivo* exposure to common recall antigens in peripheral blood (Mathew et al., 2008; Ahmadzai et al., 2012). This contrasts with the extensive local inflammation at sites of active disease and thus the phenomenon is also often referred to as an immune paradox (Miyara et al., 2006). Mechanisms of the observed sarcoid anergy are poorly understood and several theories have been proposed including compartmentalization of immune competent cells to the lung and, more recently, Treg, dendritic cells (DC), and T effector (Teff) cell dysfunction (Miyara et al., 2006; Mathew et al., 2008; Lee et al., 2011).

In fact it was initially hypothesized that the sarcoid peripheral anergy could be due to increased numbers of Treg cells that suppress proliferation of Teff cells (Miyara et al., 2006). Further studies confirmed that Treg cells are amplified in blood, BAL, and lymph node tissue of sarcoidosis patients, but these same studies indicate that these cells may in fact be impaired in their repressor function and might even contribute to pro-inflammatory granuloma formation (Taflin et al., 2009; Rappl et al., 2011; Oswald-Richter et al., 2013). Interestingly, disease resolution is associated with reversal of Th1 and Treg dysfunction (Oswald-Richter et al., 2013). Thus the original idea of Tregs contributing to peripheral anergy does not fully explain the phenomenon but nonetheless the cells seem to be dysregulated in sarcoidosis. Interestingly, peripheral blood and BAL sarcoid T-cells show selective hypersensitivity to possible sarcoid antigens while not only peripheral blood but also sarcoid BAL cells are hypo-responsive to common recall antigens (Ahmadzai et al., 2012; Oswald-Richter et al., 2013). It may be that instead of peripheral anergy, sarcoidosis is rather characterized by selective hypersensitivity to disease related antigens and hyporesponsiveness to common recall antigens. Further studies are required to compare the selective hypersensitivity and anergy in cells from BAL and peripheral blood. Likewise a role for immunosuppressive cytokines in the context of peripheral anergy in sarcoidosis should also be considered.

## IL-12 FAMILY CYTOKINES

**FIGURE 2:**
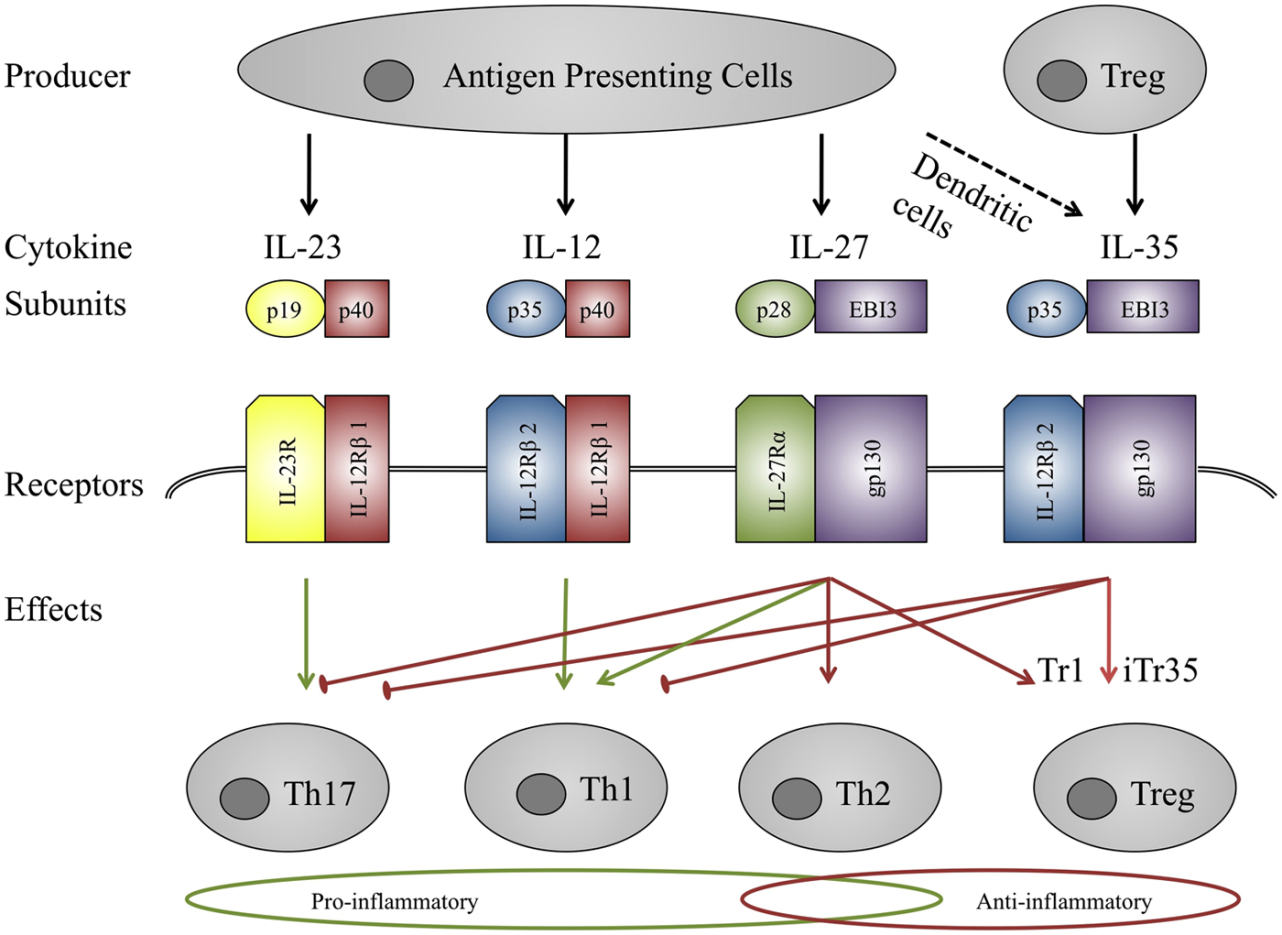
**IL-12 family members.** IL-12 family members are heterodimers sharing three α-chains (p35, p19, p28) and two β-chains (p40, EBI3). Similarly the receptors also consist of heterodimers. Signaling is mediated by the Jak-STAT family and affects different T-eff cell subsets. IL-23 and IL-12 act mainly pro-inflammatory through the activation of Th17 and Th1 cells, IL-27 seems to act in a pro-inflammatory manner on naïve T-cells and as an anti-inflammatory mediator on T-eff cells. IL-35 is immunosuppressive, inducing iTr35 cells. Green arrows indicate pro-inflammatory effects, red arrows represent anti-inflammatory properties.

Whilst the pathogenesis of granuloma formation in sarcoidosis may be at odds with observed anergy in peripheral immune cells, one possible unifying factor may be the IL-12 family of cytokines. They are a unique group of heterodimeric cytokines composed of one of the three alpha subunits p19, p35, or p28 and one of the two beta subunits p40 or Epstein-Barr virus induced gene 3 (EBI3; **Figure 2**; Vignali and Kuchroo, 2012). p40 is the shared beta-subunit of IL-12 and IL-23, whereas IL-27 and IL-35 consist of the beta unit EBI3. The alpha subunit p19 is specific for IL-23 as is p28 for IL-27, whereas p35 is shared by IL-12 and IL-35. Similarly they bind to heterodimeric receptors and share five receptor subunits. Despite their common structures the biological function of the members of this family is very diverse (**Figure 2**). Whilst IL-12 and IL-23 are pro-inflammatory, IL-27 is bi-directional in terms of being both pro- and anti-inflammatory while IL-35 is strongly immunosuppressive (Vignali and Kuchroo, 2012).

### IL-12

A role for IL-12 in sarcoid granuloma formation is well established (**Figure 1**; Iannuzzi and Fontana, 2011; Broos et al., 2013). Multiple studies confirm that IL-12p40 is elevated in blood, BAL, and granulomatous tissue from sarcoidosis patients and PBMC as well as BAL cells stimulated with sarcoid associated antigens produce higher amounts of IL-12 p40 in patients compared to healthy controls (**Table 1**). The corresponding IL-12 receptor chain IL-12Rβ1 is equally over-expressed in peripheral blood and BAL of sarcoidosis patients (Rogge et al., 1999; Taha et al., 1999; Judson et al., 2012). IL-12 is known to increase IFNγ production and this holds true in sarcoid patients compared to controls (Shigehara et al., 2001). Recent studies suggest that Th17 cells may also produce IFNγ in the presence of IL-12 (Annunziato et al., 2007; Boniface et al., 2010). IFNγ has been shown to play a pivotal role in lung granuloma formation in murine models of tuberculosis (Cooper et al., 1997). It is thus likely that IL-12 (through promoting IFNγ) is of similar importance in sarcoidosis granuloma formation.

**Table 1:** Studies on IL-12 and IL-12 receptors in sarcoidosis.

Peripheral blood:		
Method		Result	Reference

ELISA		IL-12 p40 increased, IL-12p70 not detected (ELISA).	Shigehara et al. (2003),Hata et al. (2007)
Antibody detection		IL-12Rβ2 not detected.	Rogge et al. (1999)
mRNA, rtPCR		IL-12Rβ1 is elevated compared to healthy controls, but IL-12Rβ2 is not.	Judson et al. (2012)
**Granuloma lesions:**		
**Affected organ**	**Method**	**Result**	**Reference**

Skin	mRNA, rtPCR	IL-12p40, IL-12Rβ1 and IL-12Rβ2 are elevated compared to controls.	Judson et al. (2012)
Myocardium	IL12 ELISA and mRNA	IL-12 is elevated (antibody not specified). IL-12p40 and IL-12p35 mRNA are present but only IL12p40 significantly increased compared to dilated cardiomyopathy controls.	Terasaki et al. (2008)
Lung	Immunohistochemistry	IL12p70 is overexpressed by epithelioid cells and macrophages as well as giant cells.	Shigehara et al. (2001)
Lymph node	mRNA, rtPCR	IL-12p40 mRNA and IL-12Rβ2 mRNA is elevated, but not IL12p35 or IL-12Rβ1	Hata et al. (2007)
Lung and lymph node	Immunohistochemistry	IL-12p40 is expressed by epithelioid cells and macrophages.	Shigehara et al. (2003)
Lymph node	mRNA	IL-12p40 is significantly increased compared to controls but not IL-12p35.	Bergeron et al. (1997)
**BAL:**		
**Method of detection**		**Result**	**Reference**

mRNA, ELISA		IL-12p40 mRNA and protein levels are significantly increased compared to healthy controls. BAL macrophages express higher levels of IL-12p70 both unstimulated and stimulated.	Moller et al. (1996)
ELISA		IL-12p40 is elevated compared to healthy controls, but not IL-12p70.	Shigehara et al. (2001), Barbarin et al. (2003)
Cytometric bead array		IL12p70 levels are significantly higher in patients compared to healthy controls.	Idali et al. (2006)
ELISA (not specified)		IL-12 (not specified) is elevated compared to healthy controls.	Kim et al. (2000), Meloni et al. (2004), Antoniou et al. (2006), Mroz et al. (2008)
FISH mRNA detection		IL-12p40 significantly elevated in patients with active sarcoidosis but not inactive sarcoidosis.	Minshall et al. (1997)
mRNA		IL-12Rβ1 and IL-12Rβ2 levels are elevated in sarcoidosis patients.	Taha et al. (1999)
Antibody detection		BAL cells of sarcoidosis patients express IL-12Rβ2.	Rogge et al. (1999)
**Stimulation assays with sarcoid antigens**		
**Stimulus**	**Cells**	**Result**	**Reference**

* P. acnes*	PBMC	IL-12p40 mRNA levels are significantly elevated in sarcoidosis patients compared to healthy controls.	Furusawa et al. (2012)
β-glucan/LPS	PBMC	Spontaneous and post-stimulation IL-12 protein levels are higher in patients compared to controls (IL 12 ELISA not further specified).	Rastogi et al. (2011)
NOD1/TLR4 ligands	BALF	IL12/23 p40 protein and mRNA levels are increased compared to healthy controls.	Rastogi et al. (2011)

*Multiple studies confirm IL-12p40 and IL-12Rβ1 up-regulation in peripheral blood, BAL, and granulomatous tissue, while the corresponding IL-12p35 is not dysregulated and IL-12p70 (the functional IL-12 protein) in most studies not detectable.*

### IL-23

IL-23 promotes the expansion and survival of Th17 cells, which have recently been linked to sarcoid granuloma formation (**Figure 1**; Broos et al., 2013). Gene analyses have revealed IL-23 receptor polymorphisms in sarcoidosis patients (Fischer et al., 2011; Kim et al., 2011). Furthermore IL-23 receptor mRNA is elevated in the granulomatous skin lesions of sarcoidosis patients and the same study also observed a trend for up-regulation of IL-23 p19 in two thirds of sarcoid skin lesions, yet not in peripheral blood (Judson et al., 2012). Upon *ex vivo* stimulation with toll-like receptor 9 agonists, PBMCs of sarcoidosis patients produce less IL-23 compared to healthy controls suggesting that IL-23 might play a role in the formation of granulomas but not in peripheral blood (Veltkamp et al., 2010). Given the fact that IL-23 mostly acts as a pro-inflammatory cytokine through the promotion of Th17 development, these findings underline a possible role of Th17 cells in sarcoidosis. There is evidence that the Th17 cytokine IL-17A is required in granuloma formation following infection with mycobacteria (Fitzgerald et al., 2013). Since there are to date no murine models of sarcoidosis it is difficult to verify whether Th17/IL-17A contribution is essential in sarcoidosis as well. Yet IL-23 might promote IL-17A production by Th17 cells and thus contribute to pulmonary granuloma formation.

### IL-27

Somewhat uniquely, IL-27 has been shown to have both pro- and anti-inflammatory effects (**Table 2**). Early studies focused on pro-inflammatory effects as IL-27 was shown to initiate clonal expansion of naïve T-cells and enhance INFγ production together with IL-12 (Pflanz et al., 2002). It was also found to induce Th1 differentiation and showed pro-inflammatory effects on monocytes (Owaki et al., 2005, 2006; Kalliolias and Ivashkiv, 2008). In contrast to those findings, IL-27 receptor deficient mice that are infected with *Toxoplasma gondii* can still develop an immune response but are then unable to down-regulate that response, which ultimately proves to be fatal (Villarino et al., 2003). This suggests a pivotal role of IL-27 in immune modulation and one mechanism by which this is effected may be IL-10 induction in Th1, Th2, Th17, and Treg cells. These effects seem to be mediated via both the STAT1 and STAT3 proteins (Stumhofer et al., 2007). IL-27 also promotes IL-10 – producing regulatory type 1 T-cells (Treg1) and can directly suppress Th17 cells (Apetoh et al., 2010; Sweeney et al., 2011; Fitzgerald et al., 2013). These results suggest a bidirectional function of IL-27, and while it seems to induce a pro-inflammatory response in naïve cells, the opposite is the case in activated cells (Kalliolias and Ivashkiv, 2008; Fitzgerald et al., 2013).

**Table 2:** Recent findings on pro- and anti-inflammatory effects of recombinant IL-27 on human cells in *ex vivo* studies.

Stimulated cells	Finding	Reference
**Pro-inflammatory**		
Human monocytes	Pro-inflammatory response in resting human monocytes. TLR responses are enhanced in the presence of IL-27.	Kalliolias and Ivashkiv (2008)
Human APC	Induction of pro-inflammatory CXCL10 and enhancement of TLR responses. Inhibition of LPS and CD40L mediated IL-10 production.	Zeitvogel et al. (2012)
Aplastic anaemia patients: human bone marrow mononuclear cells.	IFNγ and TNFα induction in bone marrow mononuclear cells.	Li et al. (2011)

**Anti-inflammatory**		
Human PBMC	IL-27 upregulates IL-10 production in activated PMBCs.	Fitzgerald et al. (2013)
Human PBMC	Suppression of Th17 cell development and IL-17 production in the presence of IL-23.	Fitzgerald et al. (2013)
Human DC	IL-23 enhancement after stimulation with zymosan/IFNβ is suppressed by IL-27.	Sweeney et al. (2011)

*Murine studies on IL-27 suggest a bidirectional function of the cytokine in terms of pro- and anti-inflammatory immune modulation. Recent *ex vivo* studies on human cells indicate that IL-27 might enhance inflammation in resting cells but inhibit immune responses in activated cells.*

IL-27 and STAT3 were not dysregulated in sarcoid skin granulomas whereas STAT1 and STAT3 mRNA levels were elevated in the peripheral blood of sarcoidosis patients compared to healthy controls, but another group detected co-expression of the IL-27 subunits EBI3 and p28 (**Figure 2**) in epithelioid and multinucleate granuloma cells in sarcoid lymph nodes, suggesting a role of IL-27 in granuloma formation or resolution (Larousserie et al., 2004; Judson et al., 2012). Interestingly IL-27R-/- mice produce less IFNγ at sites of granuloma formation in tuberculosis mice models suggesting that IL-27 might similarly contribute to early stages of granuloma formation in sarcoidosis (Pearl et al., 2004). At the same time IL-27 inhibits the production of TNF-α and IL-12 in activated peritoneal macrophages, suggesting an IL-27-mediated regulation of inflammation directed by macrophages in a murine model of tuberculosis (Li et al., 2012). The cytokine has recently been shown to be chemotactic for human DC and to impair HLA Class I antigen presentation in those cells (Morandi et al., 2014) and as the latter is believed to cause granuloma formation, IL-27 might thus also alter antigen presentation in sarcoidosis. IL-27 is a promising candidate for immune regulation in sarcoidosis but further studies are required to confirm IL-27 dysregulation in sarcoid tissue, analyse IL-27 expression in BALF and blood, and determine the effects of the cytokine in sarcoidosis and clarify whether it promotes inflammation and granuloma formation or contributes to disease clearance through its anti-inflammatory properties.

### IL-35

IL-35 is the most recently identified member of the cytokine family. It seems to be mainly expressed upon stimulation and there is some evidence for human Treg cells as well as DC to be a source of this cytokine (Seyerl et al., 2010; Li et al., 2012; Guttek and Reinhold, 2013). IL-35 is so far believed to be strictly immunosuppressive, mediating regulatory B- and T-cell function and increasing IL-35 – induced regulatory T-cells which express IL-35 (iTr35), as well as inhibiting effector T-cell proliferation and Th17 development and function (Niedbala et al., 2007; Collison et al., 2010, 2012; Olson et al., 2013; Ye et al., 2013; Wang et al., 2014). There are only a few studies on the role of IL-35 in human diseases and disease models to date and unlike the other IL-12 family members there are currently no studies on IL-35 in sarcoidosis. In murine models of airway inflammation, IL-35 suppressed airway hyperresponsiveness via IL-17 suppression and is elevated in BAL upon treatment with erythromycin suggesting a role for IL-35 in ameliorating airway inflammation (Bai et al., 2012; Whitehead et al., 2012). Similarly transfer of iTr35 as well as Treg cells can cure experimental inflammatory bowel disease in mice, but not if those Treg cells lack either p35 or EBI3 (Collison et al., 2007, 2010). Sarcoidosis often presents as airway inflammation and non-caseating granulomas are a feature shared with Crohn’s disease, an inflammatory bowel disease. Since Treg cell dysfunction and Th17 cells have also recently been linked to the pathogenesis of sarcoidosis IL-35 is an interesting target for further research in sarcoidosis that might contribute to disease clearance or the observed peripheral anergy.

## CONCLUSION AND PERSPECTIVES

The etiology and pathogenesis of sarcoidosis remain enigmatic. Current concepts assume that the disease is caused by exposure to disease related antigens in genetically susceptible individuals. Although sarcoidosis was initially believed to be a Th1/IL-12/IFNγ mediated disease, more recent advances have also revealed a contribution of pro-inflammatory IL-23 and Th17 cells in granuloma formation. But little is known about IL-12 family members and their possible role in fibrosis development or peripheral anergy. Whilst both IL-12 and IL-23 seem to be crucial pro-inflammatory players in granuloma formation, the only randomized controlled trial to date does not show efficacy of IL-12 and IL-23 blockade with ustekinumab in sarcoidosis (Judson et al., 2014). Given the fact that IL-12 enhances IFNγ which then promotes granuloma formation the monoclonal IFNγ antibody fontolizumab is another promising drug in the treatment of sarcoidosis showing some effects in phase 2 clinical trials in Crohn’s disease (Hommes et al., 2006). The bidirectional IL-27 has also been detected in granulomas but further studies are required in order to determine whether it enhances inflammation or downregulates the excessive immune response at sites of inflammation.

However, since both IL-35 and IL-27 also have immunosuppressive properties further studies on a potential contribution to disease remission of these immunosuppressive cytokines could shed more light on the pathogenesis of sarcoid-related fibrosis and anergy. A clearer immunosuppressive function may indicate a role for stimulation of these cytokine and their receptors in the treatment of sarcoidosis and they could also be potential diagnostic markers for the disease.

## Conflict of Interest Statement

The authors declare that the research was conducted in the absence of any commercial or financial relationships that could be construed as a potential conflict of interest.
